# Surface vs. deep acting in EFL instruction: mediating and moderating roles of emotion regulation and intelligence in burnout

**DOI:** 10.3389/fpsyg.2025.1611941

**Published:** 2025-11-12

**Authors:** Feng Zhang

**Affiliations:** College of Foreign Languages, North China University of Water Resources and Electric Power, Zhengzhou, Henan, China

**Keywords:** EFL instructor in China, teacher burnout, emotion regulation, emotional intelligence, deep and surface acting, emotional labor

## Abstract

**Introduction:**

Teaching English as a Foreign Language (EFL) is a profession characterized by high cognitive and emotional demands, leading to persistent challenges with teacher burnout (TB). While emotion regulation (ER) is known to protect against burnout, this study investigates the distinct roles of emotional labor strategies—surface acting and deep acting—within high-pressure academic cultures. It further examines whether ER mediates the relationship between surface acting and burnout, and if emotional intelligence (EI) moderates this pathway by buffering the negative effects of surface acting.

**Methods:**

A cross-sectional survey was administered to 448 EFL instructors in China, yielding a recovery rate of 93.08%. We employed structural equation modeling (SEM) to test five hypotheses, conducting mediation analysis to assess the role of ER and moderation analysis to evaluate the protective effect of EI.

**Results:**

The results confirmed that frequent use of surface acting was positively associated with higher levels of TB, whereas deep acting was linked to lower burnout. ER functioned as a significant mediator, partially explaining the detrimental impact of surface acting on burnout. Furthermore, emotional intelligence served as a significant moderator; instructors with higher EI were less affected by the negative consequences of surface acting on their wellbeing.

**Discussion:**

These findings underscore the critical distinction between dysfunctional (surface acting) and functional (deep acting) emotional labor strategies in EFL contexts. The study promotes deep acting as a more sustainable ER strategy and highlights the protective role of EI. Consequently, targeted training programs designed to enhance ER skills and develop EI are recommended as effective interventions to safeguard the wellbeing of EFL instructors operating in high-stress academic environments.

## 1 Introduction

Teaching English as a foreign language (EFL) is both a cognitively complex and emotionally demanding profession, particularly in culturally nuanced plus academically high-pressure cultures, such as China and South Korea. EFL instructors in these cultures are expected to balance language instruction with cultural sensitivity, classroom management, and emotional needs of learners (Li et al., 2024). This balancing act intensifies as EFL instructors need to incorporate enthusiasm and composure, often regardless of their internal emotional states. This challenge places them at increased risk of psychological strain and teacher burnout (TB), which, in turn, prompts the growing scholarly interest on how emotional competencies, particularly emotion regulation (ER), emotional labor (EL), and emotional intelligence (EI), impact EFL instructors' emotional wellbeing and professional growth (Rughoobur-Seetah, 2024).

One key construct in this emotional ecosystem is ER that refers to the ability of monitoring and evaluating emotional responses for meeting situational demands (Zhang and Fathi, 2024). In EFL instruction, ER is particularly crucial due to the emotionally intensive nature of the profession. Like, EFL instructors frequently face complex classroom dynamics, cultural diversity, and linguistic barriers, which can trigger emotional strain. ER enables teachers to stay composed during emotionally charged classroom interactions effectively and maintain a sense of professionalism (Fuentes-Vilugrón et al., 2024). Moreover, instructors possessing well-developed ER skills can more effectively manage interpersonal relationships across the academic community, comply with institutional expectations, and respond adaptively to pedagogical obstacles. In contrast, a lack of ER can leave instructors vulnerable to emotional dysregulation, thereby leading to reduced teaching efficacy and eventual burnout (Sadoughi et al., 2024).

Another central dimension of EL is surface acting (SA), which refers to the efforts to suppress genuine emotions and instead display emotions that are considered professionally appropriate (Alabak et al., 2023). The SA is particularly crucial in teaching profession, because instructors are expected to maintain expressions of cheerfulness, composure, or empathy, even when they fail to feel these emotions. Thus, SA can lead to emotional dissonance, which in turn increases stress, fatigue, and ultimately contributes to teacher burnout (Yikilmaz et al., 2024). Particular in high-pressure cultures, the utility of SA tends to be more frequent and socially reinforced, thus intensifying its negative impact. Besides, SA limits authentic social interactions, and often results in the feelings of emotional detachment among teachers (Pinkawa and Dörfel, 2024).

In contrast, many researchers promote a more adaptive form of EL, namely deep acting (DA), where individuals modify their internal feelings to align with outward expressions (Qiu et al., 2024). They point out that unlike SA, DA involves efforts to genuinely feel the emotions, which one is expected to display and subsequently results in greater emotional congruence plus authenticity (Mann et al., 2024). Teachers, who engage in DA, are more likely to experience sincere interpersonal interactions and enhanced emotional wellbeing. Numerous articles connect DA to increased engagement of learners along with more positive classroom dynamics. Thus, the DA appears to function as a protective factor against burnout, while also facilitating a deeper sense of meaning in work and reinforcing a strong professional identity among instructors (Hu et al., 2024).

Beyond the direct effects of EL strategies, this study finds that SA can gradually deplete an individual's capacity of controlling emotions. This scenario can make this harder to manage future emotional challenges. Instructors, who frequently engage in SA, usually experience regulatory fatigue, where sustained emotional control becomes difficult, thereby increasing their vulnerability to burnout (Tang and Gu, 2024). However, there are very few articles that examined ER as a mediator between SA and TB. Additionally, teachers with higher EI are better equipped to handle emotional demands and adapt to stress. EI supports faster recovery from emotional strain through DA, while buffering the effects of SA with enhanced emotional awareness. By utilizing their EI to establish a positive classroom climate, EFl instructors can effectively reduce their susceptibility to burnout (Iuga and David, 2024), thereby motivating this study to examine its moderating role in between SA and TB.

Therefore, despite increasing attention to burnout and emotional wellbeing, this study finds many professional development initiatives to overlook the central role of emotional skills. Institutional support for managing emotional challenges is often very few, and policies aimed at improving teacher retention often fail to address the psychological realities of classroom work (Brandão et al., 2024). Moreover, existing articles are largely situated in Western contexts, with insufficient exploration of the emotional dynamics faced by EFL instructors in culturally distinct nations, like China (Wang et al., 2024). In this regard, this study seeks answers to the following research questions:

- This study considers the relationship between the ability to regulate emotions and the degree of TB among EFL instructors in China. This leads to the research question: to what extent is ER negatively associated with TB?
- This study examines how how instructors' reliance on SA, while altering outward expressions without changing internal states, contributes to emotional dissonance and heightened TB, thus asking: how does SA influence TB among EFL instructors?
- This study explores whether the instructors, who rely more on DA (by modifying internal feelings to match external expressions) experience lower TB than those who rely much on SA, thereby prompting this study to ask the following: How does the impact of DA on TB compare to that of SA among EFL instructors?
- This study can identify whether SA indirectly contributes to TB through its negative impact on ER. In other words, does ER mediate the relationship between SA and TB among EFL instructors?
- This study also examines whether higher EI can buffer the negative impact of SA on TB. Thus, this study determines the following aspect: does EI moderate the relationship between SA and TB in such a way that this relationship is weaker among instructors with higher EI?

This way, the present study plans to determine actionable insights for diverse stakeholders with a focus on preventing burnout, while strengthening ER in instructional contexts, and developing both the professional development and overall wellbeing of EFL instructors.

The rest of this paper begins with Section 2 providing a brief review of recent literature in current context. Next, Section 3 frames five major hypotheses and two contextual lemmas on basis of review of established literature. Then, Section 4 illustrates the research methodology of this study, whereas Section 5 performs a number of statistical tests over current research data. Subsequently, Section 6 conducts the validation tests for the proposed hypotheses and thereby extracts a number of implementable insights for various stakeholders. Section 7 provides the conclusions of this study, and discusses some limitations of this study along with associated future research scopes.

## 2 Literature review

This section provides the review of recent and established literature on various aspects of current research as follows:

### 2.1 Burnout in teaching profession

TB has become an increasing concern in educational institutions worldwide, particularly due to its significant impact on both the teachers themselves and the quality of education they provide. Defined by Maslach and Jackson's three-dimensional model, burnout includes emotional exhaustion, depersonalization, and a reduced sense of personal accomplishment (Avola et al., 2025). Emotional exhaustion refers to feelings of being overwhelmed and depleted of emotional resources, while depersonalization manifests as a detached or indifferent attitude toward students and colleagues (Fauzan et al., 2023). The third dimension, reduced personal accomplishment, involves a negative self-evaluation of one's competence and effectiveness in the role (Xu and Jia, 2022). The teaching profession inherently involves high levels of EL, which is often considered a major contributor to TB. EL requires teachers to regulate their emotions intentionally, frequently necessitating the suppression of personal emotions to align with professional expectations and institutional norms (Faulkner and Thompson, 2023). In educational contexts, this may involve presenting a cheerful demeanor, even when teachers feel frustrated or exhausted (Jalilzadeh et al., 2024). This continuous ER can lead to emotional dissonance, where there is a mismatch between the emotions felt and those that must be displayed, contributing significantly to burnout (Rughoobur-Seetah, 2024).

EL in teaching is particularly challenging for EFL instructors, who must often adapt their emotional expressions to accommodate the emotional needs of students while managing their own emotional exhaustion. The costs of EL in the teaching profession, both at an individual and organizational level, can result in long-term negative outcomes, including strain, stress, and eventual burnout (Pultz and Dupret, 2024). Teachers engaged in EL frequently experience frustration, exhaustion, and dissatisfaction, all of which are closely linked to higher burnout rates (Ngcobo et al., 2022). Additionally, the frequent use of ER in student interactions can be emotionally draining for teachers, thus elevating their risk of burnout and consequently impairing their professional effectiveness and personal wellbeing. Research distinguishes between two types of EL: SA and DA. SA involves feigning emotions that one does not actually feel, often leading to emotional suppression, stress, and TB (Ngcobo et al., 2022). In contrast, DA entails modifying one's internal emotional state to align with the emotions that need to be expressed, which can reduce emotional dissonance and provide emotional rewards. While SA is linked to emotional exhaustion and TB, DA has been found to have more positive psychological outcomes and may even serve as a buffer against burnout in some cases (Yang and Jang, 2022). Despite these differences, research indicates that both SA and DA have their costs in high-emotion jobs, with SA typically resulting in more detrimental effects such as emotional exhaustion (Nielsen et al., 2023).

A key challenge for EFL instructors is balancing obligatory performative EL with authentic emotional engagement. While SA meets immediate expectations, the DA provides a greater potential for improving teaching satisfaction and preventing burnout. However, most of the existing research has failed to examine these two strategies in isolation, which leaves a gap in understanding whether DA buffers the negative consequences of burnout in the context of academic environment (Nielsen et al., 2023). The consequences of burnout extend beyond emotional exhaustion and job dissatisfaction; they significantly affect teaching quality and student outcomes. Burnout is linked to a reduction in teachers' ability to engage effectively with students, which can lead to disengaged teaching, lack of motivation, and diminished job satisfaction (Ribeiro et al., 2022). Teachers suffering from burnout often experience a decrease in the quality of their work, as emotional exhaustion undermines their cognitive resources and energy, which in turn affects their teaching performance (Mir Jalalli, 2024). Moreover, burnout leads to increased absenteeism and turnover, further disrupting the educational environment and diminishing the overall quality of instruction (Chen et al., 2024).

The emotional demands of the job are particularly taxing. The constant need to manage students' emotional needs, adapt to diverse cultural contexts, and maintain personal emotional wellbeing can create a high-risk environment for burnout. The emotional exhaustion associated with these demands negatively impacts both the instructors' emotional and professional capacities, leading to a decline in teaching effectiveness and reduced job satisfaction (Vero and Dongni, 2024). In turn, these negative outcomes can also have cascading effects on students, as they may encounter less enthusiastic, disengaged, and emotionally drained instructors (Warner and Diao, 2022). To address the challenges associated with TB, this is essential to focus on coping strategies that can alleviate emotional strain. One approach is to foster emotional resilience through training programs that equip teachers with tools to manage stress and regulate their emotions effectively. Research emphasizes the importance of providing teachers with coping mechanisms, such as mindfulness, stress management techniques, and social and emotional learning initiatives (Trinidad, 2025). Furthermore, improving organizational support and creating a positive work environment are critical to mitigating the effects of burnout. Studies have shown that teachers who receive support from colleagues, administrators, and the broader school community are less likely to experience burnout (Tubei et al., 2024).

### 2.2 On emotion regulation of teachers

Researchers find the ER as a critical psychological process, which enables individuals to manage and modulate their emotional experiences and expressions in response to situational demands. This capacity is particularly essential in high-interaction professions like teaching. For EFL instructors, the constant requirement to regulate emotions is fundamental to maintain professional conduct and facilitate effective instruction (Dreer, 2024).

Thus, ER is an individual skill, and is deeply influenced by workplace norms and organizational expectations. In educational environment, teachers' ER abilities are vital for developing positive interactions with students, thus preventing emotional dissonance, and ensuring a productive and supportive learning environment (Yang and Du, 2024). In the context of teaching, ER is crucial for managing emotional demands from students, colleagues, parents, and institutional pressures. When teachers successfully regulate their emotions, they maintain a positive classroom atmosphere, which contributes to improved student outcomes and teacher efficacy (Aldrup et al., 2024). The importance of ER in enhancing workplace norms and job satisfaction is evident, as this directly influences teachers' ability to cope with the diverse challenges of teaching (Namaziandost et al., 2023). In teaching profession, where the demand for emotional control is high, the cost of ER is significant. Teachers frequently face the pressure to suppress or modify their emotional expressions in order to align with the expectations of students, institutional norms, and the broader educational community (Riforgiate et al., 2022). EFL instructors, in particular, often find themselves in situations, where their personal emotional states must be transformed to align with established norms of professionalism. Language instructors frequently face personal and emotional challenges that remain invisible in classroom environment, thus adding additional psychological burdens (Qu and Wang, 2024). These demands are compounded by the need to maintain emotional distance from students to avoid being perceived as unprofessional. Thus, teachers must develop sophisticated emotional, pedagogical, and personal strategies to manage these conflicting emotional demands.

The failure to regulate emotions adequately can have significant consequences for teachers' emotional wellbeing and professional performance. EL costs increase when employees, including teachers, are expected to display a wide range of emotions, particularly when these emotions contradict internal feelings (Elfenbein, 2023). For EFL instructors, the emotional strain from suppressing or altering emotions can lead to emotional exhaustion, burnout, and a decline in teaching quality. Without proper ER, teachers are more likely to become overwhelmed by stress, negatively affecting their teaching performance, relationships with students, and overall wellbeing. Therefore, ER is an essential tool for maintaining a healthy work environment and ensuring long-term professional success (Xiao and Tian, 2023). ER strategies can broadly be categorized into adaptive and maladaptive strategies. Adaptive ERS involve effective emotional control that aligns with the demands of the situation, helping individuals maintain positive emotional states while meeting situational needs. Maladaptive ERS, on the other hand, involve ineffective methods of emotional control that typically result in negative emotional outcomes, such as increased stress, anxiety, and TB (Navas-Casado et al., 2023). Teachers who employ maladaptive ERS are likely to experience emotional dissonance, leading to diminished job satisfaction, poor work performance, and an increased risk of burnout.

Researchers show the importance of adaptive ER strategies and demonstrate that teachers, who effectively regulate their emotions, report enhanced wellbeing, increased job satisfaction, along with improved classroom performance. Also, they experience less emotional exhaustion and greater professional fulfillment, which in turn enhances the quality of teaching (Mutlu and Solhi, 2024). Conversely, teachers who struggle with ER face significant challenges in managing the emotional demands of teaching, which can lead to long-term burnout and reduced effectiveness in the classroom. Despite the critical role that ER plays in teacher wellbeing, research on ER among higher education teachers, particularly EFL instructors, remains relatively scarce. The emotional demands placed on teachers are multifaceted and complex. EFL instructors, in particular, face additional pressures related to cultural and linguistic diversity, which further exacerbate their emotional workload (Seis, 2023). These instructors are often required to manage not only the emotional needs of students but also their own emotional responses to challenging teaching situations. Effective ER serves as a buffer against the negative psychological outcomes associated with EL, such as burnout (Gadassi-Polack et al., 2024). Teachers who use adaptive ERS are better equipped to manage stress, maintain positive relationships with students, and sustain their emotional wellbeing over time. Given the unique emotional demands of teaching, educational institutions need to support teachers in enhancing their ER skills (Bai and Li, 2025).

### 2.3 On emotional labor of teachers

EL refers to the intentional regulation and management of emotions within the workplace, often in interactions with colleagues, students, or clients. In educational environment, particularly in teaching English, EL is a crucial component that shapes teacher-student relationships and the overall teaching dynamic. Teachers are frequently required to regulate their emotions to create a productive learning environment, often without acknowledging the emotional toll this can take on their wellbeing (Faulkner and Thompson, 2023). Given that teaching is inherently interactive, managing emotions effectively is vital for sustaining job satisfaction and professional efficacy (Jalilzadeh et al., 2024). EL theory, introduced by Arlie Hochschild, emphasizes the intentional suppression or modulation of emotions to meet organizational expectations. Bolton and Ronnagil expanded this framework, highlighting the diverse emotional expressions that may arise in professional environment, including teaching (Pultz and Dupret, 2024). The degree of ER required can vary in educational contexts, depending on factors such as the teacher's role, the organizational culture, and the characteristics of the students and the environment (Young et al., 2024).

Understanding ER is essential for teachers, as this helps them control their emotional responses in various teaching situations, preventing these emotions from negatively impacting themselves or their students. ER is especially important in teaching, where emotional interactions with students are frequent and intense. Teachers must manage personal emotions such as frustration, fatigue, and stress, while maintaining a professional and supportive demeanor despite the emotional challenges they face (Rughoobur-Seetah, 2024). The high emotional demands of the profession, particularly in diverse classrooms, can lead to substantial EL costs. If these costs are not managed, they can contribute to burnout and reduced job satisfaction.

Within the concept of EL, two primary strategies are commonly discussed: SA and DA. SA involves the outward display of emotions that do not align with the individual's internal emotional state. For example, a teacher may be required to display enthusiasm or warmth, even when feeling frustrated or exhausted. This form of EL often leads to emotional suppression, which can contribute to burnout, fatigue, and decreased performance over time (Ngcobo et al., 2022). Conversely, DA involves not only the outward display of emotions but also an internal attempt to align one's true feelings with the emotions that need to be expressed. Teachers engaged in DA work to internalize and genuinely experience the emotions they project, which can result in emotional authenticity and greater satisfaction in the role (Nesher Shoshan and Venz, 2022).

However, research has consistently shown that SA, particularly under conditions of high work pressure, increases emotional dissonance and emotional fatigue, which are precursors to burnout (Yang and Jang, 2022). While DA may mitigate some of the negative effects of SA, this requires significant emotional effort, which, if not managed properly, could also lead to emotional exhaustion over time. Therefore, understanding the balance between surface and DA, as well as the circumstances under which each type of EL is employed, is essential for promoting a sustainable and healthy teaching environment.

The impact of EL on teachers extends far beyond their personal wellbeing and emotional health. This influences their student engagement and job satisfaction (Blake and Dewaele, 2023). Teachers are expected to model ER for their students, thereby influencing the emotional climate in classrooms (Smith et al., 2025). In the context of foreign language teaching, this challenge is even more pronounced, as teachers must navigate the emotional complexities of assisting students in acquiring a new language while also managing cultural nuances and their own emotional responses.

### 2.4 Research gaps

Although the emotional demands of teaching and its relationship with burnout have been extensively studied, this study finds that significant gaps remain, especially concerning EFL instructors. While existing literature identifies EL as a key predictor of TB (Jalilzadeh et al., 2024; Smith et al., 2025), there are very few articles that systematically examine how SA and DA differentially affected TB among EFL instructors in an academically demanding nation (Yang and Jang, 2022; Nesher Shoshan and Venz, 2022).

Moreover, this study finds that many articles describe ER as crucial in managing teaching-related stress and maintaining emotional balance (Stellern et al., 2023; Avola et al., 2025), even while its mediating role in the relationship between SA and TB are rarely explored. Also, EI has been associated with better stress management and job satisfaction (Xue, 2022), even though there are very few empirical researches that have tested its moderating role in buffering the impact of SA on TB.

## 3 Framing the hypotheses

Current review of established literature prompts this study to frame the following hypotheses:

### 3.1 Impacts of emotion regulation on burnout of EFL instructors

Teaching is an emotionally demanding profession that requires instructors to manage their emotions effectively while developing a productive learning environment. This emotional demand is particularly pronounced for EFL instructors, who must simultaneously deliver language instruction, manage classroom behaviors, and navigate the diverse cultural and linguistic backgrounds of their students. In these high-pressure environments, ER becomes a crucial skill that enables instructors to maintain composure, sustain motivation, and cope with the emotional complexities of their profession (Landa and English, 2022). ER involves a set of cognitive and behavioral strategies aimed at modifying individual's emotional responses to external stimuli. In this regard, some common approaches include cognitive reappraisal (CR), where an individual alters their interpretation of a situation to change their emotional reaction, and expressive suppression (ES), where outward emotional expressions are inhibited (Gross and John, 2003).

Many articles discuss that burnout among teachers, especially EFL instructors, stems from prolonged work-related stressors like excessive workload, EL, and continuous student interaction. Emotional exhaustion depletes their resources and often leads to fatigue and disengagement, whereas depersonalization worsens burnout by weakening teacher-student interactions. Effective ER prevents burnout by providing adaptive coping strategies. CR, in particular, enables teachers to reframe stress constructively, thus reducing distress and building resilience. By managing emotions effectively, instructors can minimize emotional dissonance and reduce associated internal conflict, thereby decreasing levels of exhaustion and preventing professional disengagement (Ergül Bayram and Eveyik-Aydın, 2023). Moreover, the continued development of a supportive work environment can strengthen ER, which ultimately helps EFL instructors to maintain their wellbeing and professional commitment in more sustainable manner.

Among strategies related to controlling emotions, several articles describe DA to be particularly effective in reducing burnout. Unlike SA, which involves faking emotions to meet professional expectations, DA encourages instructors to genuinely align their internal emotions with their outward expressions. This authentic emotional engagement lowers stress and prevents emotional fatigue. Teachers, who practice DA, build stronger connections with their student through meaningful interactions that boost motivation and reduce depersonalization (Alabak et al., 2023). Also, ER helps instructors to develop emotional resilience, thus enabling them to handle challenges without succumbing to burnout. Those with strong ER skills can recognize early signs of stress and apply strategies, such as mindfulness and peer support, to mitigate its effects. These proactive approaches reduce burnout risk and boost job satisfaction. In contrast, instructors with poor ER skills are more prone to chronic stress, which leads to emotional exhaustion and diminished effectiveness (Nesher Shoshan and Venz, 2022). Consequently, this study frames the following hypothesis:

*H*_1_**:** Higher levels of emotion regulation are negatively associated with burnout among EFL instructors.

### 3.2 Impacts of surface acting on burnout of EFL instructors

Researchers find that teachers face intense challenges as they navigate language instruction, student engagement, and diverse cultural dynamics. To meet professional expectations, instructors frequently rely on regulation strategies to maintain composure and effectively respond to classroom demands (Landa and English, 2022). One prevalent form of ER is SA, where instructors modify their outward emotional expressions without altering their internal emotional state. Instructors engaging in SA suppress their true emotions while displaying emotions that align with institutional expectations, often masking fatigue, frustration, or disengagement with forced enthusiasm (Gross and John, 2003).

Despite its short-term effectiveness in maintaining a professional demeanor, SA has long-term psychological costs. Researchers describe that the emotional dissonance resulting from portraying inauthentic emotions places significant strain on instructors, thus leading to emotional exhaustion, which is a core component of TB (Wang, 2022). SA requires continuous cognitive and emotional effort to suppress true feelings, which depletes psychological resources over time. The inability to express genuine emotions can also create a sense of depersonalization, where instructors feel detached from their work, thus exacerbating burnout symptoms (Ergül Bayram and Eveyik-Aydın, 2023).

The prolonged use of SA limits the opportunities for authentic emotional engagement with students and reduces overall job satisfaction. Although some instructors attempt to mitigate burnout through DA, which involves modifying inner emotions to align with external expressions, this study finds SA to be more taxing due to the persistent internal conflict this generates (Alabak et al., 2023). For EFL instructors, who often face high emotional demands from students with varying linguistic abilities and cultural backgrounds, the pressure to engage in SA can intensify emotional fatigue. As a result, the cumulative burden of SA contributes to heightened levels of TB over time (Nesher Shoshan and Venz, 2022). While SA can temporarily guide to manage professional expectations, its prolonged use intensifies emotional exhaustion, thus ultimately exacerbating burnout. Therefore, this deliberation leads the present study to frame one hypothesis as follows:

*H*_2_**:** Higher levels of surface acting are positively associated with burnout of EFL instructors.

### 3.3 Deep acting as protective factor against burnout than surface acting

Many articles determine that EFL instructors regularly engage in ER to navigate the challenges of teaching. However, the way they regulate their emotions—whether through DA and/or SA, plays a crucial role in determining their levels of burnout. Like, SA frequently produces emotional dissonance, a misalignment between an instructor's internal feelings and their outward emotional expressions (Hu et al., 2024). This persistent mismatch creates significant psychological strain, as teachers must constantly suppress their genuine emotions, thereby leading to heightened stress, fatigue, and emotional exhaustion. Over time, SA depletes an instructor's emotional resources, which make this difficult to sustain engagement, satisfaction, and motivation in the classroom. Consequently, instructors, who predominantly rely on SA, experience a higher risk of burnout due to the continuous demand to regulate emotions in a way that feels inauthentic (Yikilmaz et al., 2024).

In contrast, DA enables instructors to modify their internal emotional states to align with expected expressions. Thus, instructors can develop a more authentic emotional experience (Alabak et al., 2023). Rather than merely suppressing or faking emotions, DA allows teachers to genuinely connect with their students and cultivate positive emotions that enhance the teaching experience. This emotional alignment reduces cognitive and psychological strain, while preventing the emotional exhaustion commonly associated with SA. Besides, DA brings meaningful teacher-student relationships, as students are more likely to perceive their instructors' emotions as sincere (Nesher Shoshan and Venz, 2022). This positive classroom environment reinforces the instructor's sense of purpose and fulfillment, while creating intrinsic motivation that helps counteract TB. By promoting emotional authenticity, the policy of employing DA develops greater emotional control and resilience, and thus enables instructors to manage stress more effectively.

Subject to these contrasting effects, this study emphasizes that instructors, who predominantly engage in DA, experience significantly lower burnout levels compared to those who frequently rely on SA. While both strategies require effort, SA imposes an additional psychological burden by fostering emotional dissonance, whereas DA promotes emotional alignment and authenticity, thus making this to be a more sustainable and effective approach (Mann et al., 2024). The ability to internalize and genuinely experience required emotions through DA enhances instructors' emotional wellbeing, job satisfaction, and professional longevity. Thus, this study considers a hypothesis that promoting DA over SA through training programs and institutional policies can be a crucial step in reducing burnout and enhancing overall instructional effectiveness in EFL contexts, as follows:

*H*_3_**:** EFL instructors, who predominantly rely on deep acting, experience lower levels of burnout compared to those, who frequently use surface acting.

### 3.4 Emotion regulation as mediator between surface acting and burnout

Researchers describe that ER has a critical role in determining how EFL instructors cope with the emotional demands of teaching, particularly when they engage in SA (LI and AKRAM, 2023). Notably, SA creates a discrepancy between true feelings and displayed emotions. This emotional dissonance places significant strain on instructors, and brings difficulty to manage emotions effectively. SA gradually weakens instructors' ER abilities by consistently suppressing their natural emotional responses and hindering the development of adaptive coping strategies like CR or emotional distancing (Zhang and Wang, 2024). As a result, instructors, who rely heavily on SA, can experience a decline in their ability to regulate emotions constructively.

The mediating role of ER between SA and TB is reflected in the management of emotional resources. When instructors engage in SA, they expend significant effort maintaining a professional facade that conflicts with their true emotions (Yikilmaz et al., 2024). This effort is mentally draining and diminishes the cognitive capacity needed for effective ER. Moreover, instructors, who struggle with ER due to excessive SA, find this harder to employ adaptive strategies, like problem-focused coping or emotional detachment. As a result, they usually experience increased stress and lower job satisfaction. Thus, SA indirectly contributes to burnout by impairing ER, which demonstrates that the more an instructor suppresses emotions through SA, the harder this becomes to regulate their emotions in a healthy and adaptive way (Hu et al., 2024).

Thus, this study finds that strengthening ER skills is essential for mitigating burnout risks among EFL instructors. When instructors possess well-developed ER skills, they are better equipped to manage the stressors of SA, while reducing its adverse effects on burnout. Institutions can provide targeted training programs on ER approaches, such as mindfulness, cognitive restructuring, and emotional detachment, to help instructors develop healthier coping mechanisms (Landa and English, 2022). By enhancing ER strategies, educational institutions can break the cycle in which SA leads to burnout, and instead promote emotional resilience and wellbeing of EFL instructors. Consequently, this study frames one hypothesis as follows:

*H*_4_**:** Emotion regulation mediates the relationship between surface acting and burnout such that increased reliance on surface acting undermines effective emotion regulation, thus leading to higher burnout among EFL instructors.

### 3.5 Emotional intelligence as moderator between surface acting and burnout

The EI moderates the extent to which SA leads to emotional exhaustion and depersonalization. Although SA is inherently linked to burnout due to the emotional dissonance this usually generates, EFL instructors with high EI can more effectively mitigate the psychological strain resulting from this dissonance (Chen et al., 2024). Specifically, EI enables individuals to recognize, understand, and regulate emotions effectively, thus reducing the long-term emotional depletion that SA typically induces. As a result, although SA generally leads to burnout, its adverse effects are significantly less pronounced among instructors with higher EI (Li and Zhang, 2024).

The moderating role of EI can be explored by investigating how instructors with varying levels of EI manage EL. Many articles find out that high-EI instructors are more adept at employing CR strategies, which allow them to reinterpret stressful interactions in a way that minimizes emotional distress (Sariraei et al., 2024). Also, their superior self-regulation skills enable them to maintain psychological equilibrium even when engaging in SA, thus preventing the accumulation of emotional fatigue. This emotional resilience reduces the likelihood of experiencing burnout despite frequent engagement in SA. In contrast, low-EI instructors lack these adaptive coping mechanisms (Li and Zhang, 2024), which makes them more vulnerable to the negative consequences of SA. Without the ability to regulate emotions effectively, they experience prolonged emotional strain, which accelerates burnout, thus motivating this study to hypothesize the following:

*H*_5_**:** Emotional intelligence moderates the positive association between surface acting and burnout, which is weaker with higher emotional intelligence of EFL instructors.

### 3.6 Moderating role of emotional regulations: cognitive reappraisal and expressive suppression

The contemporary literature consistently demonstrates that sustained reliance on SA intensifies emotional exhaustion and diminishes the professional efficacy of instructors. Building on this evidence, the present study first hypothesizes a direct relationship between SA and TB. However, existing literature also indicates that the strength of this association is hardly uniform across individuals; rather shaped by personal and contextual factors. In particular, the magnitude of the relationship between SA and TB is conditioned by the ER strategies that instructors adopt to navigate affective demands in their professional roles. Within this scenario, this study focuses on two central ER sub-dimensions, namely CR and ES, which diverge substantially in their underlying cognitive processes and implications for instructors' wellbeing [ref]. Considering these dynamics, this seems essential to examine their moderating influence in succession, thereby determining whether instructors' preferred regulation styles mitigate or amplify the heightened risk of TB associated with SA.

In this regard, the CR refers to an adaptive strategy, which involves the reinterpretation of potentially distressing situations in ways that alter their emotional impact [ref]. By reframing emotionally challenging classroom encounters, instructors usually plan to reduce the subjective strain of displaying organizationally prescribed emotions, thereby weakening the detrimental effect of surface acting. Also, the reappraisal is associated with more positive affect, lower physiological arousal, and improved interpersonal outcomes [ref]. In educational institutions, instructors, who are high in reappraisal, are more likely to perceive emotionally charged interactions with students or parents as manageable, which in turn reduces the likelihood of emotional depletion. Accordingly, apart from aforementioned hypotheses, this study frames the following lemma:

*L*_1_**:** Cognitive reappraisal moderates by weakening the positive association between surface acting and burnout among EFL instructors, thereby serving as a protective emotion regulation approach.

In contrast, ES is defined as the inhibition of outward emotional expressions without modifying the underlying emotional experience. Thus, ES has been widely characterized as a maladaptive regulatory strategy. Even though suppression can provide some short-term benefits like maintaining classroom order, researchers find its long-term consequences to be less adaptive. Those are linked to heightened physiological activation, diminished emotional authenticity, and impaired interpersonal functioning [ref]. Thus, any habitual reliance on suppression can intensify the dissonance between experienced and displayed emotions, which consequently compound the strain already induced by SA of instructors. Over time, this incongruence is also likely to increase exhaustion and detachment, thus elevating the risk of burnout. Thus, this study frames the following lemma:

*L*_2_**:** Expressive suppression moderates the positive relationship between surface acting and teacher burnout such that this becomes stronger among instructors with higher levels of suppression.

**Figure 1:**
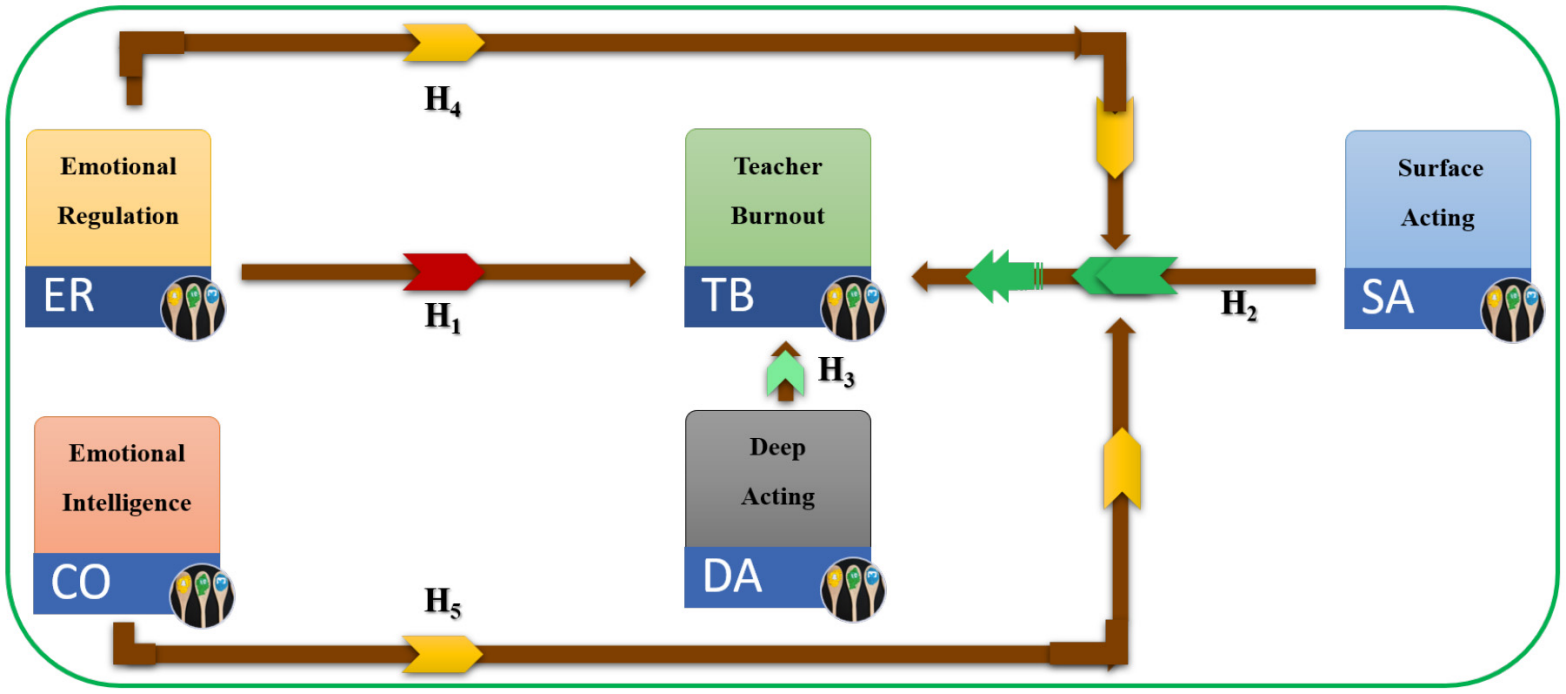
Hypothesized relationships among constructs of this study.

Figure 1 describes the graphical interconnection among current constructs.

## 4 Research methodology

This section firstly considers the participants of a primary data-based survey to measure various constructs of this study, while also describing the associated scales in this regard.

### 4.1 Participants

The present study employed a rigorous random sampling strategy to ensure diversity and representativeness across Chinese EFL instructors, while initially reaching out to 417 EFL instructors from eight provinces in China. The survey was conducted between August 2024 to January 2025, and encompassed varied institutional and regional contexts. After data screening and removal of incomplete and/or inconsistent responses, the final research data comprised 448 EFL instructors as participants, with a high success rate of 93.08%. The respondents ranged in age from 24 to 56, and also represented various genders, educational backgrounds, and research interests, and had teaching experience spanning three to over 25 years. Notably, the participants were drawn from multiple schools and higher education institutions, which broadened representativeness of dataset.

This survey process relied on established online platforms, including WeChat, Wenjuanxing, SoJump, and Xuexi Tong, to maximize reach and convenience. Detailed instructions were provided to ensure clarity and ease of participation, whereas the survey included a structured questionnaire that was carefully pre-tested with a small sample for reliability and validity before distribution. The participants received detailed instructions that guided them through the survey process. They were allowed to complete the questionnaire at their own pace, with the flexibility to return and finish responses in multiple sessions if needed. Moreover, to enhance response rates, platform-based follow-up reminders were sent to reduce non-response and attrition. These measures collectively strengthened participation rates and improved the quality of responses.

Ethical considerations were central to the design of this survey. Participants provided informed consent electronically before beginning the survey, with clear documentation outlining the purpose of this study, the voluntary nature of participation, and the right to withdraw at any stage without penalty. Anonymity and confidentiality were rigorously maintained, with all data securely stored and used exclusively for research purposes. This study adhered to international ethical standards and institutional review board guidelines, thus ensuring that participants' rights and wellbeing were prioritized throughout. Specifically, the participants were seasoned EFL instructors, and thus could bring a wealth of expertise to this study. Also, they could adapt instructional strategies to diverse student needs.

Moreover, the reliance on self-reported data introduced the possibility of common method bias and response bias, which are recurring challenges in any survey-based educational research. This study minimized those risks through multiple procedural remedies. Like, the assurances of anonymity and confidentiality reduced tendencies toward socially desirable responding. Neutral wording, randomized item presentation, and balanced scale designs were used to reduce acquiescence and consistency biases for Chinese EFL instructors. In addition, the pre-testing phase refined the questionnaire to avoid ambiguous or leading questions, thereby enhancing interpretability and accuracy. Participants were reminded that there were no correct or incorrect answers, which further encouraged authentic responses. Despite all these precautions, the possibility of residual CMB and response bias would remain. This prompted the present study to employ the Harman's single-factor test during the analysis phase to statistically assess the extent of CMB. Results indicated no dominant single factor, and thus established that CMB was unlikely to pose a threat to the validity of current insights.

### 4.2 Research instruments

This study utilizes a number of well-established scales to assess ER, TB, SA, DA, and EI. These scales are essential for understanding the psychological and emotional dynamics instructors experience, particularly in high-stress environments, such as the classroom. The use of these well-validated scales ensures the reliability and validity of current findings.

In particular, this study applies the popular emotion regulation questionnaire (ERQ) scale (Gross and John, 2003) to assess ER, while specifically focusing on reappraisal and suppression strategies. The ERQ scale consists of 10 items, with responses rated on a 7-point Likert scale, ranging from 1 (strongly disagree) to 7 (strongly agree). Notably, reappraisal involves changing the way one thinks about a situation to alter its emotional impact, while suppression involves inhibiting emotional expressions. Thus. this scale plays a key role in measuring how individuals regulate their emotions, which is crucial for managing stress and maintaining wellbeing. Higher reappraisal scores typically indicate more adaptive ER, while higher suppression scores are often associated with negative outcomes, such as heightened stress and burnout. Also, this study uses first, third, fifth, seventh, eighth, and tenth columns in responses to measure CR, whereas this utilizes the rest four columns of responces to determine the ES.

To measure TB, this study uses the Maslach burnout inventory-educators survey (MBI-ES) scale (Maslach and Jackson, 1981), which refers to a widely used tool that can assess burnout in high-stress professions. The MBI-ES scale in this study consists of 22 items, while being rated on a 7-point Likert scale, ranging from 0 (never) to 6 (every day). This scale measures three key dimensions of burnout, namely emotional exhaustion, depersonalization, and reduced personal accomplishment. Emotional exhaustion reflects feelings of being emotionally drained, depersonalization involves detachment from students, and reduced personal accomplishment refers to a diminished sense of competence. Next, this study employs the emotional labor scale (ELS) (Brotheridge and Lee, 2003) to assess the emotional demands placed on EFL instructors. The ELS consists of 6 items, rated on a 5-point Likert scale, ranging from 1 (never) to 5 (always). This scale measures both SA and DA. Notably, SA is linked to negative outcomes, such as emotional dissonance and increased stress, while DA is seen as a more adaptive strategy that leads to better job satisfaction and lower emotional strain.

Then this study employs the popular Wong and Law emotional intelligence scale (WLEIS) (Wong and Law, 2002) to measure EI. The WLEIS consists of 16 items, rated on a 7-point Likert scale, ranging from 1 (strongly disagree) to 7 (strongly agree). This scale evaluates the respondents' ability to perceive, evaluate, and regulate their own emotions as well as the emotions of others, whereas any higher scores on EI have been associated with better coping strategies, improved interpersonal relationships, and lower stress levels. Thus, current scales provide a comprehensive assessment of constructs among EFL instructors in highly-stressful teaching environments.

### 4.3 Data validation with control variables

Ensuring data validation is crucial for maintaining the accuracy and reliability of this study. This study carefully integrates control variables to address factors that can influence the relationships among constructs, namely ER, TB, SA, DA, and EI. The control variables used in this analysis include demographic factors, such as age, gender, and years of teaching experience, all of which are known to affect ER, burnout, and coping strategies. By incorporating these control variables, this study mitigates potential confounding effects, thereby isolating the true effects of the primary constructs under investigation and improving the precision of the findings. These demographic factors are included in the analysis alongside the primary independent and dependent variables, which can ensure that the observed relationships are as accurate and unbiased as possible.

Here, the relationships between ER, SA, DA, EI, and TB are minimally influenced by external factors, which enhances the internal validity of the results. To ensure data quality and reliability, this study employs a range of established statistical tools and methods. Like, Cronbach's α test (CAT) is utilized to assess the internal consistency of the scales used in the study, whereas skewness and kurtosis analyses are performed to examine the distribution of the research data, thus ensuring that the data meets the assumptions necessary for subsequent analyses. Also, the Kaiser-Meyer-Olkin (KMO) measure and Bartlett's sphericity test (BST) are conducted to confirm the adequacy of the current research data for factor analysis. To further strengthen the validity of the study, exploratory factor analysis (EFA) is performed to identify any underlying constructs that shall influence the relationships between the main variables. This test is followed by advanced statistical techniques, including correlation analysis, regression analysis, and mediation analysis, to examine the complex relationships among ER, SA, DA, EI, and TB. These statistical methods are particularly effective for incorporating control variables, enhancing causal inference, and improving model fit.

In particular, the SEM analysis should provide strong evidence for the relationships among the primary constructs, while controlling for the demographic factors. The mediation analysis, which investigates the role of ER as a mediator between SA and TB, and the moderation analysis regarding the role of EI, can contribute to the understanding of the mechanisms that drive burnout among EFL instructors. Along with the moderation analysis, the present study shall perform a Johnson-Neyman significance region technique, which allows for the examination of the interaction between SA and EI in predicting TB. This method is particularly useful in identifying specific levels of EI at which the relationship between SA and burnout becomes statistically significant. Notably, the Johnson-Neyman technique should provide a detailed picture of the moderation effect. By integrating control variables into the statistical analysis and employing advanced moderation techniques such as the Johnson-Neyman significance region, current comprehensive approach can ensure that the relationships observed are robust and valid.

## 5 Results on current research data

This section reports the results of various statistical tests over current research data while then analyzing those as follows:

### 5.1 Demographic characteristics

This study provided the major demographic characteristics of the 417 EFL instructors who participated in the current survey. Table 1 provided this information, which found that the proportion of female EFL instructors constituted 46.68% (195), while male teachers accounted for 53.32% (222). This balanced gender distribution provided a fair representation of both male and female teachers, thus ensuring that any gender-based variations in ER and/or burnout among EFL instructors could be effectively analyzed.

**Table 1:** Demographic characteristics for EFL instructors (*n* = 417).

**Demographic variable**	**Percentage (%)**	**Frequency (n)**
**Gender**		
Male	53.32	222
Female	46.68	195
**Age group**		
24–30 years	15.90	66
31–40 years	35.61	149
41–50 years	29.98	125
51–56 years	18.51	77
**Years of experience**		
5–10 years	21.6	90
11–15 years	30.2	126
16–20 years	25.9	108
21–25 years	13.8	58
More than 25 years	8.6	35
**Educational qualification**		
Masters	51.91	217
Bachelors	35.0	146
Ph.D.	17.10	71
**Research interest**		
Applied linguistics	38.8	162
Linguistics	34.5	144
TESOL	26.7	111

Also, this study found that the age distribution among the EFL instructors varied, with the largest age groups being 35.61% (149) for 31 − 40 years and 29.98% (125) for 41 − 50 years, whereas the age groups of 24–30 years and 51–56 years were represented by 15.90% (66) and 18.51% (77) of current research data. This diversity in age among respondents allowed for a broader understanding of how ER and coping mechanisms could differ across different life stages, from early-career teachers to those with more extensive experience. Regarding years of experience, the EFL instructors in the sample exhibited a wide range of tenure in the profession. The largest group consisted of teachers with 11 − 15 years of experience (30.2%, 126 participants), followed by those with 16–20 years (25.9%, 108 participants), 5 − 10 years (21.6%, 90 participants), 21 − 25 years (13.8%, 58 participants), and more than 25 years (8.6%, 35 participants). This variation in experience levels would be essential for understanding how the duration of teaching experience could influence TB and EL.

In terms of educational background, more than half (51.91%, 217) of the respondents held a master's degree, while 35.0% (146) had a bachelor's degree, and 17.10% (71) possessed a doctorate. This educational distribution indicated that the current research data consisted predominantly of well-educated teachers, with a substantial proportion holding advanced degrees. Consequently, their diverse academic backgrounds contributed to varied perspectives on ER and ERS in the classroom, as well as their ability to reflect on and report their emotional responses to stressors. Moreover, this study found that the research interests of the EFL instructors were diverse. The majority of participants were interested in applied linguistics (38.8%, 162 participants), followed by those with an interest in linguistics (34.5%, 144 participants) and TESOL (26.7%, 111 participants). This diversity implied a broad range of scholarly fields of interest for the EFL teaching community.

Notably, the demographic tendencies of current research dataset indicated certain imbalances in age and education levels of Chinese EFL instructors, even while this study could adequately reflect their prevailing profile. In particular, the concentration of participants in the mid-career age range of 31–50 years implied that the proposed insights would primarily reflect the perspectives of EFL instructors, who had already established professional routines and coping strategies, whereas the voices of both novice instructors and late-career educators were comparatively less visible. Again, the predominance of participants with master's and doctoral qualifications indicated that the results were shaped largely by highly educated instructors. Thus, this dataset failed to fully represent the experiences of EFL instructors holding only bachelor's degrees. One should recognize these demographic imbalances as potential influences on the observed patterns of ER, EL, and EI.

### 5.2 Reliability analysis

This study utilized the CAT values to evaluate the reliability and internal consistency of current research data. Table 2 displayed that CAT values for all constructs in this study were >0.7 and thus confirmed high reliability with great internal consistency, thereby ensuring the appropriateness of the scales used. Specifically, ER had the highest CAT value of 0.960, which demonstrated excellent internal consistency. This result implied that the items used to assess ER were strongly correlated and effectively captured the underlying construct of ER. Also, TB scale exhibited a CAT value of 0.958, which reinforced its reliability in measuring the burnout levels of EFL instructors. The high reliability score suggested that the TB scale successfully encapsulated the various dimensions of teacher burnout, thus ensuring consistent responses across items.

**Table 2:** Results of reliability analysis along with KMO, BST, and factor loading for constructs (*n* = 417).

**Item**	**KMO**	**BST χ^2^**	**df**	***p*-value**	**CAT**	**Skewness range**	**Kurtosis range**	**AFL range**
ER	0.963	4,668.675	45	0.000	0.960	[–1.958, –0.129]	[–1.264, 3.556]	[0.288, 0.577]
TB	0.941	7,110.581	231	0.000	0.958	[–4.611, –1.817]	[2.288, 25.133]	[0.186, 0.547]
SA	0.881	1,186.047	15	0.000	0.879	[–5.524, –3.998]	[15.760, 38.687]	[0.157, 1.051]
DA	0.815	655.236	6	0.000	0.839	[–5.619, –3.759]	[14.587, 38.687]	[0.158, 0.836]
EI	0.748	4,292.791	120	0.000	0.906	[–8.967, –4.984]	[22.837, 92.149]	[0.081, 0.344]

ER, emotion regulation; TB, teacher burnout; SA, surface acting; DA, deep acting; EI, emotional intelligence; AFL, average factor loading.

Current results showed that SA and DA achieved CAT values of 0.879 and 0.839, respectively. While both values met the acceptable reliability standards, this study determined that SA displayed a slightly higher internal consistency compared to DA. This finding suggested that the items measuring SA were more cohesively structured, whereas the variability in responses for DA could reflect individual differences in DA strategies among instructors. Despite this slight variation, both scales remained reliable measures of surface and deep forms of acting. Additionally, the EI scale yielded a CAT value of 0.906, which confirmed its strong internal consistency. This finding indicated that the items used to assess EI were highly interrelated and captured the construct effectively. The high reliability of EI supported its moderating role in the relationship between SA and TB, thus ensuring that the construct was measured with precision. Therefore, the high CAT values of various constructs indicated well-designed questionnaire items, while consequently ensuring reliable and interpretable analyses.

Next, this study computed skewness and kurtosis values of constructs. In particular, the skewness value for ER ranged from –0.1293 to –1.9576, which meant mostly high levels of ER with some variability. The corresponding kurtosis values, ranging from –1.2643 to 3.5563, showed varying degrees of peakedness with some items exhibiting concentrated responses. For TB, the skewness ranged from –1.8169 to –4.6108, thus indicating that most instructors reported higher burnout, particularly at extreme levels. Its kurtosis values (2.2877 to 25.1329) indicated that burnout responses were highly concentrated around extreme values, which could reinforce the widespread nature of TB among EFL instructors. The skewness values for SA ranged from –3.9213 to –5.5243 to indicate a frequent use, whereas its kurtosis values (15.7601 to 38.6874) advocated for extreme concentration of responses. This finding showed the dominant role of SA in EL of Chinese EFL instructors.

Likewise, DA showed negative skewness (–3.7594 to –5.6192) and high kurtosis (14.5871 to 38.6874), thereby indicating that DA was also widely adopted but with a concentrated distribution. EI exhibited the most extreme negative skewness (–4.9837 to –8.9673). Whereas these values for EI indicated high levels of EI among most instructors, its kurtosis values (22.8373 to 92.1490) showed a highly concentrated distribution, which reinforced the moderating role of EI in buffering burnout. Therefore, the negative skewness values indicated that instructors generally reported high levels of emotional demands, while the extreme kurtosis values showed that EL and burnout were common and intense, thereby particularly supporting the mediation effect of ER in the SA-burnout relationship (hypothesis *H*_4_) and the moderating effect of EI (hypothesis *H*_5_).

### 5.3 KMO and BST tests along with factor loading

Table 2 displayed that the KMO values of various constructs of this study ranged from 0.748 to 0.963. Specifically, ER showed the highest KMO value of 0.963 to confirm excellent sampling adequacy for factor analysis, whereas a high KMO value of 0.941 for TB exhibited the suitability for factor extraction. Also, the KMO values for SA (0.881) and DA (0.815) remained well above the acceptable threshold of 0.7, thereby confirming their appropriateness for factor analysis. Nevertheless, EI could have the lowest KMO value of 0.748, which was above the minimum threshold of 0.7, and thus validated the adequacy of the sample for factor analysis. This way, the present study established that all constructs displayed strong sampling adequacy, and supported the use of factor analysis for examining their underlying structures.

Moreover, the BST results in Table 2 validated the suitability of current research data for factor analysis, as all constructs exhibited highly significant *p*-values. Notably, ER had a χ^2^ value of 4,668.675 with a *p-*value of 0.000, whereas TB recorded the highest χ^2^ value of 7110.581 with a *p*-value of 0.000. These values confirmed strong inter-item correlations for both constructs. Likewise, SA showed a χ^2^ value of 1,186.047, while DA had a χ^2^ value of 655.236, both with *p*-values of 0.000, thereby ensuring the factorability of these constructs. Despite EI presenting the lowest KMO value, its χ^2^ value of 4,292.791 and *p*-value of 0.000 strongly supported its inclusion in factor analysis.

Regarding the EFA results, this study revealed that the factor loadings for ER ranged from 0.2884 to 0.5772, which indicated items having moderate associations with the underlying construct. Despite the moderate range, the consistency in loadings suggests acceptable representation of ER. For TB, factor loadings varied between 0.1864 and 0.5474, thus reflecting low to moderate associations and suggesting variability in item performance. The SA showed factor loadings from 0.1569 to 1.0513 to indicate a wide range from weak to strong associations. Thus, while some items strongly captured SA, others could have been less aligned conceptually. Moreover, factor loadings for DA ranged from 0.1581 to 0.8360, which showed that most items had moderate to strong associations with the factor. EI had factor loadings between 0.0807 and 0.3442, and thus implied relatively weak item-factor associations.

### 5.4 Correlation analysis

This study conducted the correlation analysis to examine the relationships among constructs on current research data. Table 3 displayed the matrix to advocate for a strong positive correlation between ER and TB (0.8516). This finding suggested that ER was used as a coping strategy, even while this tool could hardly protect EFL instructors from burnout. SA had a high correlation with TB (0.8878), which confirmed the significant contribution of SA to burnout and thus supported the proposed Hypothesis *H*_2_. Also, this study found that DA was strongly correlated with TB (0.8421). Thus, DA, typically referred as a typical adaptive strategy, failed to fully prevent burnout of EFL instructors in China.

**Table 3:** Correlation matrix of constructs (*n* = 417).

**Construct**	**ER**	**TB**	**SA**	**DA**	**EI**
ER	1	0.852	0.657	0.608	0.543
TB		1	0.888	0.842	0.785
SA			1	0.868	0.864
DA				1	0.897
EI					1

Moreover, this study found that ER correlated positively with SA (0.6573) and DA (0.6085). These values indicated that the EFL instructors, who could regulate their emotions, were more likely to engage in both EL strategies. The high correlation between SA and DA (0.8679) indicated that instructors often employed both strategies simultaneously. Besides, EI correlated positively with SA (0.8641), DA (0.8973), and TB (0.7855), thus implying that EI influenced TB to a lesser extent. Therefore, current correlation matrix confirmed significant relationships among constructs, while providing strong preliminary support for the proposed hypotheses and showing the complexity of EL in teaching a foreign language.

### 5.5 Harman's single-factor test

The present study conducted the Harman's single-factor test to assess the potential threat of CMB. The results, reported in Tables 4, 5 indicated that the first unrotated factor accounted for 48.86% of the total variance, which is below the conventional threshold of 50%. This implied that no single factor dominated the variance structure of current research data, and thus CMB was unlikely to be a serious concern. The distribution of variance across multiple factors also confirmed that the observed relationships among ER, SA, DA, EI, and TB could hardly be inflated artificially by measurement artifacts. Thus, the Harman's single-factor test results provided confidence in the validity of subsequent hypothesis testing for the proposed hypotheses *H*_1_−*H*_5_.

**Table 4:** Results of Harman's single-factor test (*n* = 417).

**Test component**	**Variance explained (%)**	**Threshold**
First factor variance	48.86	< 50 (No CMB risk)
Total variance explained	100.0	—
Threshold flag (CMB risk if >50%)	False	—

**Table 5:** Variance explained by additional factors.

**Factor**	**Variance (%)**	**Factor**	**Variance (%)**	**Factor**	**Variance (%)**	**Factor**	**Variance (%)**
2	7.432	4	2.929	6	2.386	8	1.940
3	3.774	5	2.569	7	2.319	9	1.768
10	1.638	factors 11–58: < 1.5 each					

### 5.6 Structural equation modeling

The results of SEM analysis revealed several significant path estimates that contributed to a deeper understanding of how these factors interact. Table 6 provided the results, and thus demonstrated a direct and significant positive effect of SA on TB, with a path coefficient of 0.1607 (β= 0.160715, *p* < 0.001). This finding suggested that increased reliance on SA was linked to higher levels of burnout. Also, ER had a positive effect on burnout among EFL instructors (β = 0.1447, *p* < 0.001), which indicated that better ER could help instructors manage burnout, despite some level of burnout resulting from emotional demands. DA had a stronger positive effect on burnout (β = 0.6298, *p* < 0.001), thus supporting the role of emotional effort in contributing to burnout. Besides, the path estimates showed that ER significantly influenced SA with a coefficient of 0.7619 (β = 0.7619, *p* < 0.001). Thus, this study implied that higher levels of ER were associated with increased SA of EFL instructors.

**Table 6:** Results of structural equation modeling for constructs (*n* = 417).

**Path/Index**	**Estimate**	**Standard error**	**z-value**	***p*-value**	**Note**
**Structural paths**					
ER ~ SA	0.7619	0.0441	17.264	< 0.001	
TB ~ ER	0.1447	0.0057	25.331	< 0.001	
TB ~ SA	0.1607	0.0112	14.299	< 0.001	
TB ~ DA	0.6298	0.0483	13.036	< 0.001	
TB ~ SA × EI	0.0140	0.0017	7.990	< 0.001	interaction
ER ↔ ER (var.)	0.8122	0.0562	14.440	< 0.001	residual
TB ↔ TB (var.)	0.0111	0.0008	14.440	< 0.001	residual
**Fit indices**	**Value**	**Fit indices**	**Value**	**Fit indices**	**Value**
degrees of freedom	8	χ^2^	44.83	*p*-value	< 0.001
χ^2^ baseline	2377.59	CFI	0.984	GFI	0.981
AGFI	0.969	NFI	0.981	TLI	0.975
RMSEA	0.105	AIC	13.78	BIC	42.02
Log likelihood	0.108				

Current results found that ER significantly mediated the relationship between SA and TB. The path from SA to ER (β= 0.7619, *p* < 0.001) was significant, which implied that SA influenced ER. The positive effect of ER on TB (β= 0.1447, *p* < 0.001) contributed to the overall mediation effect, whereas its total effect through ER supported that greater reliance on SA would reduce effective ER and would lead to higher burnout levels. This finding supported the key role of ER in mitigating burnout, even though SA directly contributed to higher burnout levels.

Nevertheless, the path involving the interaction between EI and SA on TB was statistically non-significant, despite EI showing a direct positive influence on burnout, with a coefficient of 0.01396 (β = 0.013959, *p* < 0.001). This finding suggested that EI would play a key role in mitigating burnout through other mechanisms. Regarding model fit, the SEM analysis yielded strong results. The chi-square value (χ^2^ = 44.828, *p* < 0.001) was statistically significant, while the CFI of 0.9844 and GFI of 0.9811 were excellent. The Root RMSEA value of 0.105 was acceptable for behavioral research models, whereas AIC and BIC values supported the robustness of the model. Thus, this study provided valuable insights into the emotional demands faced by EFL instructors and the roles that ER and EI would play in managing teacher burnout in China.

### 5.7 Mediation analysis

The results of mediation analysis in Table 7 provided substantial support for the mediating role of ER in the relationship between SA and TB among EFL instructors in China. In particular, this study found that the path from SA to ER (path a) was statistically significant, which could confirm that instructors engaged more frequently in SA exhibited lower levels of ER. Also, the path from ER to TB (path b) demonstrated a significant relationship, thus indicating that a reduction in ER would contribute to increased TB. Moreover, this relationship was supported by the bootstrapped mediation results, which revealed a significant indirect effect (β = 0.4944), with a 95% confidence interval of [0.4196, 0.5750]. These findings indicated that ER partially mediated the relationship between SA and TB. Therefore, EFL instructors, who relied on SA, faced difficulties in ER, which led to higher levels of burnout.

**Table 7:** Results of bootstrapped mediation analysis of constructs (*n* = 417).

**Path**	Indirect effect (a*b)	**95% CI**
SA → ER → TB	0.4950	[0.4154, 0.5848]

Additionally, the presence of a significant indirect effect indicated that while SA directly influenced TB, a portion of this effect was transmitted through ER. The indirect pathway illustrated that SA, which often involved suppressing true emotions in favor of display rules, depleted instructors' ability to regulate their emotions effectively. This depletion, in turn, led to emotional exhaustion and, consequently, higher levels of burnout. Despite the indirect effect being significant, this study could find that the direct effect of SA on TB remained notable. Thus, ER od EFL instructors in China played a mediating role, whereas their SA independently contributed to TB. This way, current findings advocated for the role of ER in reducing the negative impact of SA on burnout among EFL instructors. Instructors with effective ER managed teaching demands better, while those relying on SA without ER faced higher burnout risks. All these aspects confirmed ER as a crucial buffer against the negative effects of SA, thereby supporting the proposed hypothesis *H*_4_.

### 5.8 Moderation analysis using the Johnson-Neyman significance region

**Figure 2:**
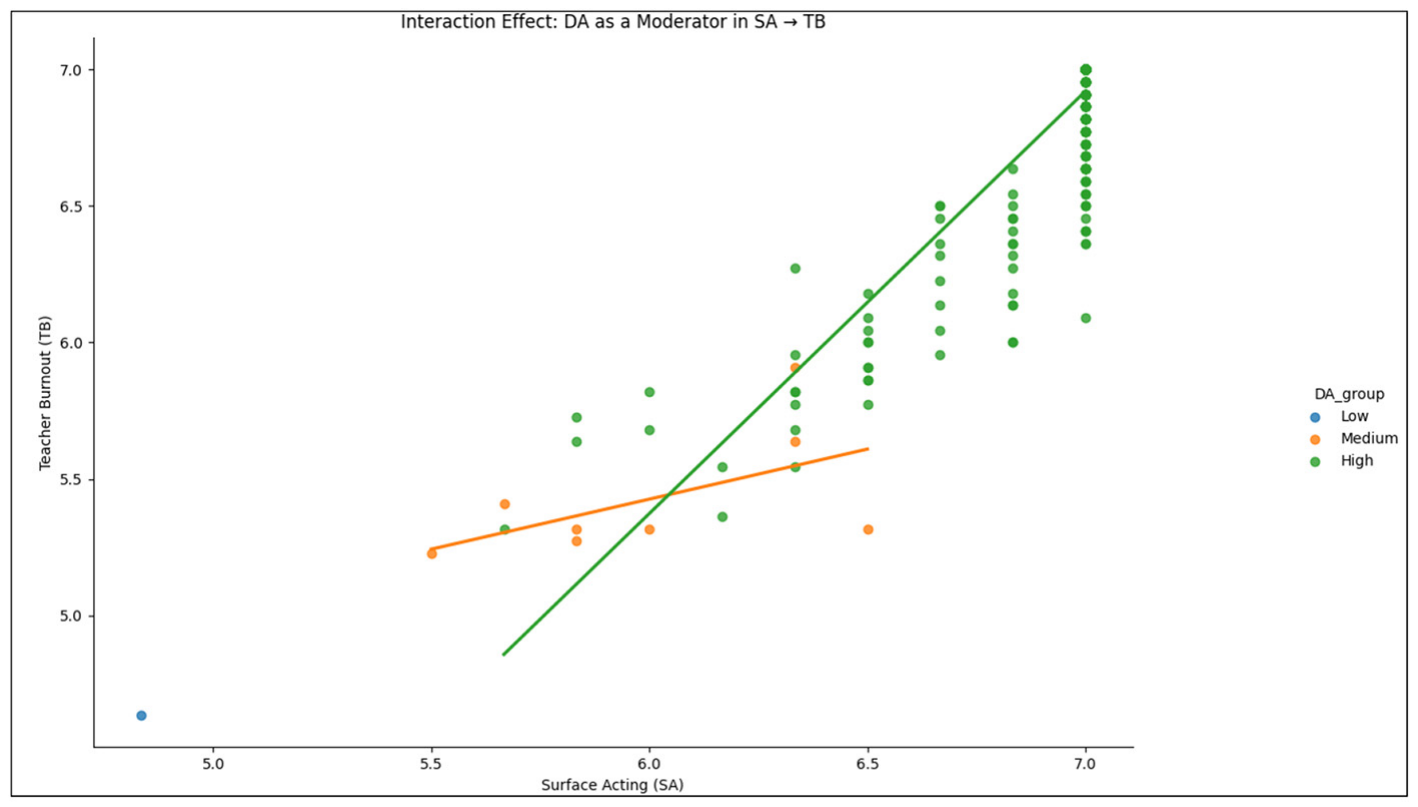
Interaction effect of DA between SA and TB of EFL instructors.

**Figure 3:**
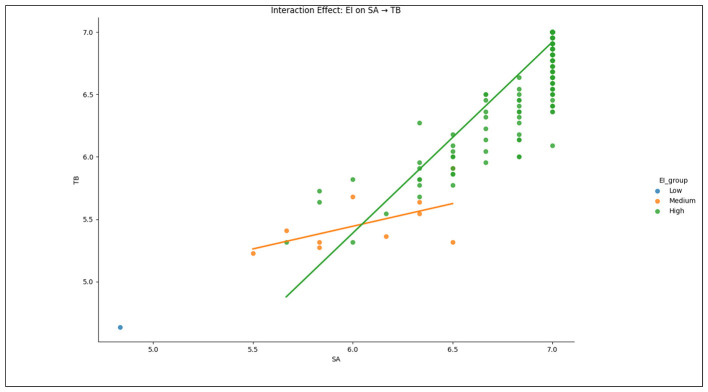
Interaction effect of EI between SA and TB of EFL instructors.

The moderation analysis provided compelling evidence for the moderating roles of EI and DA in the relationship between SA and TB. The results in Tables 8–11 along with Figures 2, 3 demonstrated that EI significantly moderated the positive association between SA and TB (β = 1.0569, *p* < 0.001), which could confirm that the adverse effects of SA on burnout were less pronounced for instructors with higher EI. Also, this moderation effect suggested that emotionally intelligent instructors possessed the ability to manage the emotional strain associated with SA, thereby mitigating its impact on burnout. In this regard, the Johnson-Neyman significance region for EI ranged from –1.1255 to 0.0279, thus indicating a significant moderating effect only within this interval.

**Table 8:** Results of moderation analysis with EI as moderator for SA → TB (*n* = 417).

**Predictor**	**Coefficient**	**Standard error**	***t*-value**	***p*-value**	**95% CI**
SA	1.3087	0.064	20.603	0.000	[1.184, 1.434]
EI	1.4646	0.183	8.013	0.000	[1.105, 1.824]
Interaction (SA * EI)	1.0569	0.104	10.123	0.000	[0.852, 1.262]

Johnson-Neyman significance region for EI: [–1.13, 0.03].

**Table 9:** Results of quadratic moderation analysis with EI as quadratic moderator of SA → TB (*n* = 417).

**Predictor**	**Coefficient**	**Standard error**	***t*-value**	***p*-value**	**95% CI**
SA	1.6279	0.086	18.851	0.000	[1.458, 1.798]
EI	0.7076	0.228	3.103	0.002	[0.259, 1.156]
Interaction (SA * EI)	2.4472	0.283	8.662	0.000	[1.892, 3.002]
EI^2^	–2.5361	0.481	–5.271	0.000	[–3.482, –1.590]

**Table 10:** Results of curvilinear moderation analysis (*n* = 417).

**Predictor**	**Coefficient**	**Standard error**	***t*-value**	***p*-value**	**95% CI**
SA	1.5617	0.083	18.720	0.000	[1.398, 1.726]
EI	2.5611	0.372	6.887	0.000	[1.830, 3.292]
SA * EI	4.0092	0.371	10.810	0.000	[3.280, 4.738]
EI^2^	0.7020	0.699	1.004	0.316	[–0.672, 2.076]
SA * EI^2^	2.1763	0.353	6.159	0.000	[1.482, 2.871]

**Table 11:** Results of moderation analysis with DA as moderator for SA → TB) (*n* = 417).

**Predictor**	**Coefficient**	**Standard error**	***t*-value**	***p*-value**	**95% CI**
SA	1.1363	0.060	19.044	0.000	[1.019, 1.254]
DA	0.9356	0.073	12.857	0.000	[0.793, 1.079]
SA * DA	0.5505	0.045	12.212	0.000	[0.462, 0.639]

Johnson-Neyman significance region for DA: [–1.73, 0.07].

This way, this study found a significant curvilinear pattern in the moderating effect of EI. Specifically, EFL instructors possessing EI levels that exceeded a identified upper threshold, hardly experienced any statistically significant attenuation of the SA-TB relationship. This indicated that the protective buffering capacity of EI against the deleterious effects of SA was subject to a limit of efficacy. Beyond this threshold, the mitigating influence of EI appeared to plateau, which implied that even high levels of this competency failed to fully insulate individuals from the cumulative strain of persistent emotional trouble. These results provided robust empirical confirmation for the nuanced relationship postulated in hypothesis *H*_5_.

Also, the curvilinear moderation model showed the non-linear interaction between SA and EI in predicting TB. The interaction term (SA*EI) showed a strong positive relationship with TB (β = 4.0092, *p* < 0.001), and also the quadratic interaction term was significant (β = 2.1763, *p* < 0.001). These findings meant that the moderating role of EI followed a curvilinear pattern, where moderate levels of EI provided a protective effect against burnout. However, this buffering effect weakened at higher levels, while the non-significant effect of EI (*p* = 0.316) indicated that although EI moderated the relationship, its squared term failed to contribute. This scenario advocated for a threshold beyond which its protective effect plateaued.

Moreover, this study determined DA as a significant moderator in the relationship between SA and TB. The interaction between SA and DA (SA*DA) was highly significant (β = 0.5505, *p* < 0.001), which indicated that instructors, who were engaged in DA alongside SA, should experience lower burnout levels compared to those relying solely on SA. This result demonstrated that DA, which involved modifying internal feelings to align with expressed emotions, helped EFL instructors to alleviate the emotional dissonance caused by SA, thereby reducing burnout. Moreover, this study found that the Johnson-Neyman significance region for DA (–1.7264 to 0.0665) indicated that the moderating effect was significant within this range. Therefore, DA played a protective role in buffering the detrimental effects of SA on TB, whereas the moderation model for DA achieved a high explanatory power with an adjusted *R*^2^ of 0.859. This scenario would establish that DA significantly contributed to explain the variations in TB among EFL instructors in China.

Also, this study measured the quadratic moderation model, which indicated that the interaction between SA and EI followed a complex pattern in predicting TB. The significant negative coefficient of EI (β = −2.5361, *p* < 0.001) revealed that at extremely high levels of EI, the protective effect of EI on burnout started to decline. This result implied a non-monotonic relationship, where EI acted as a buffer against burnout at moderate levels yet became less effective at excessive levels. Besides, the strong interaction effect (SA*EI, β = 2.4472, *p* < 0.001) reinforced that the interplay between SA and EI was critical in determining burnout levels among instructors. These results strongly supported the proposed hypothesis *H*_5_, and should be effective to yield insights into how EFL instructors needed to manage the negative effects of SA across educational institutions in China.

#### 5.8.1 Moderation analysis for lemmas *L*_1_ and *L*_2_

This study conducted the moderation analysis to examine the suitability of the proposed lemmas *L*_1_ and *L*_2_ and derived as follows:

• *Lemma* *L*_1_*:* The results in Table 12 found the interaction term, SA × CR, as positive and statistically significant (β = 0.249, *p* < 0.001). This finding indicated that the CR significantly moderated the relationship between SA and TB, whereas the simple slopes analysis showed that the effect of SA on TB was positive and significant across all levels of CR, yet with varied strength. At low levels of CR (−1 SD), the effect of SA on TB was weaker (β = 1.441, *p* < 0.001), even though the effect was stronger (β = 2.151, *p* < 0.001) at high levels of CR (+1 SD). Moreover, the Johnson–Neyman test identified that the moderation effect was significant when CR ranged from −4.220 to 1.613 (centered scale). These findings validated the proposed lemma *L*_1_ by confirming that the use of CR by EFL instructors altered the intensity of the relationship between SA and TB. However, contrary to its theorized buffering role, higher CR appeared to amplify rather than mitigate the impact of SA on burnout.

**Table 12:** Results of moderation analysis for *L*_1_ and *L*_2_.

**Hypothesis**	**Interaction term (β)**	**LM (−1 SD)**	**HM (+1 SD)**	**Johnson–Neyman interval**
*L*_1_: SA × CR → TB	0.249^***^	1.441^***^	2.151^***^	−4.220 to 1.613
*L*_2_: SA × ES → TB	0.174^***^	1.064^***^	1.374^***^	−3.905 to 0.595

^***^ *p* < 0.001. LM, low moderator; HM, high moderator.

One plausible explanation for this result is the heightened cognitive load imposed by simultaneously engaging in SA and frequent reappraisal. While CR is generally adaptive, its effectiveness shall diminish under conditions of chronic emotional labor, such as prolonged SA, where reinterpreting situations demands sustained cognitive effort. Over time, this additional regulatory burden depletes the EFL instructors' mental resources and increase fatigue, thereby intensifying rather than alleviating burnout. Moreover, when reappraisal fails to substantially alter the affective experience associated with inauthentic displays, this can produce a sense of inefficacy, thus further compounding the risk of exhaustion and disengagement. Therefore, this study concludes that the CR, under persistent SA conditions, can inadvertently exacerbate the strain this was expected to buffer.

•*Lemma* *L*_2_*:* This study assessed whether the ES intensified the relationship between SA and TB. Here, the interaction term (SA × ES) was positive and significant (β = 0.174, *p* < 0.001), which supported the proposed moderation. The simple slopes analysis indicated that SA consistently predicted higher burnout at all levels of ES, even while the association became stronger as ES increased. Specifically, the effect of relationship between SA and TB was weakest at low ES (β = 1.064, *p* < 0.001) and strongest at high ES (β = 1.374, *p* < 0.001). Besides, the Johnson–Neyman analysis advocated for a significant moderation effect within the range of −3.905 to 0.595 (centered scale). These results confirmed the lemma *L*_2_, while demonstrating that EFL instructors, who relied more heavily on suppression, experienced an intensified relationship between SA and TB. Therefore, this study promoted that both forms of ER, namely CR and ES, moderated the relationship between SA and TB. Specifically, the CR could strengthen the relationship rather than buffering this, whereas the ES predictably amplified the risk of burnout associated with SA.

## 6 Validation of hypotheses and implementable insights

This section tests the validity of the proposed hypotheses and thereby extracts a number of implementable insights for various stakeholders of this study.

### 6.1 Validation of the proposed hypotheses

This study sought to validate the proposed hypotheses based on the current statistical findings. This study observed a direct effect of ER on TB for EFL instructors (estimate = 0.1447, *p* < 0.001), whereas the relationship remained statistically significant. This finding indicated that ER helped EFL instructors manage their emotions, which, in turn, reduced their burnout levels. The negative association between ER and TB implied that EFL instructors, who effectively regulated their emotions, had experienced lower burnout, likely due to better stress management. These results confirmed that ER played a protective role in minimizing burnout among Chinese EFL instructors. Thus, this study validated the proposed hypothesis *H*_1_.

Next, this study noted that the path from SA to TB was significant (coef = 1.5617, *p* < 0.001), whereas the direct effect from SA to TB also remained strong (estimate = 0.1607, *p* < 0.001). Thus, the EFL instructors, who frequently engaged in SA, exhibited higher levels of burnout. The emotional dissonance engendered by the sustained dissonance between outward emotional expressions and internal affective states significantly contributed to instructors' exhaustion. The robust positive relationship observed between SA and TB substantiates the assertion that SA depletes instructors' finite emotional resources, thereby heightening their vulnerability to burnout. Consequently, these findings provided strong empirical support for the hypothesis *H*_2_.

In this regard, this study determined that DA played a different role in influencing TB of Chinese EFL instructors, as its effect was lower than that of SA. Current results indicated that DA had a significant effect on TB (coef = 0.6298). However, the moderation analysis revealed that SA had a stronger influence on TB than DA. Thus, this study would promote that while DA contributed to TB, its impact was less pronounced compared to SA. Therefore, instructors, who were engaged in DA likely aligned their emotions with their expressions, while reducing emotional dissonance and subsequently experiencing lower burnout. The contrast in effects between SA and DA on TB confirmed that DA mitigated burnout levels relative to SA. Thus, this study validated the proposed hypothesis *H*_3_.

Then, this study found that ER mediated the relationship between SA and TB, as indicated by a significant indirect effect (a*b = 0.4965, 95% CI [0.4190, 0.5805]). This result suggested that SA negatively influenced ER, which, in turn, heightened TB. The strong regression path from SA to ER (β = 0.7619, *p* < 0.001) and from ER to TB (β = 0.1447, *p* < 0.001) further reinforced this mediation effect. This way, current mediation results implied that EFL instructors, who relied on SA struggled with ER, which could lead to increased TB. Consequently, this study provided empirical support for the mediation effect of ER in the relationship between SA and TB, thereby validating the proposed hypothesis *H*_4_.

Moreover, this study examined the moderating effect of EI on the SA-TB relationship. The interaction term for SA and EI was significant (β = 4.0092, *p* < 0.001), which could demonstrate that EI moderated this relationship. The Johnson-Neyman significance region (–1.1255 to 0.0279) indicated that the positive association between SA and burnout was weaker for instructors with higher EI, whereas the quadratic moderation model additionally supported this finding, with EI squared yielding a significant negative coefficient (β = −2.5361, *p* < 0.001). These results confirmed that Chinese EFL instructors with higher EI were less affected by the negative effects of SA on TB, thereby supporting the proposed hypothesis *H*_5_.

This way, this study provided strong empirical evidence validating the five proposed hypotheses, while advocating for the complex interplay of ER, SA, DA, and EI in predicting TB among EFL instructors.

### 6.2 Implementable insights

The present study yields several actionable insights based on current statistical findings. Educational institutions, policymakers, EFL instructors, and other stakeholders can apply the following insights to design evidence-based interventions that reduce burnout and enhance wellbeing:

• **Strengthening ER to prevent burnout of Chinese EFL instructors:**

*Current findings:* ER significantly reduces TB (*r* = −0.8516) and mediates the impact of SA on burnout (β = 0.4944, 95% CI [0.4196, 0.5750]).

*Policy recommendations for China:* This study emphasizes the need for targeted interventions to support teacher wellbeing. Specifically, this study proposes to implement training and development programs by incorporating institutional training on emotion-focused coping techniques, such as mindfulness, reappraisal, and acceptance-based strategies. ER workshops should be integrated into professional development programs to enhance teachers' ability to manage emotional demands. Also, AI-powered emotional health monitoring can be utilized, while employing AI-driven sentiment analysis tools to assess teacher wellbeing through self-reported data and speech/text patterns. This study pleads to arrange automated ER recommendations, which are based on real-time feedback of EFL instructors and can aid in ER. Moreover, incentivizing self-regulation practices can encourage EFL instructors to prioritize their mental health, with schools providing incentives, such as extra leave days and wellness rewards for those who actively engage in self-regulation practices.

*Global insights:* ER programs should be culturally adapted, like incorporating local stress-relief practices (e.g., meditation in Asia, “fika” breaks in Scandinavia).

• **Management of SA to reduce burnout of Chinese EFL instructors:**

*Current findings:* SA strongly correlates with TB (*r* = 0.8878) and directly exacerbates burnout (β = 0.1607, *p* < 0.001).

*Policy recommendations for China:* To mitigate the negative effects of SA on TB, this study pleads to reduce the need for forced emotional display. This policy provides teachers with greater autonomy over their classroom engagement styles. Like, they can modify lesson delivery methods to encourage authentic teacher-student interactions. Emotional support systems, including free psychological counseling and peer support groups, should be implemented to equip teachers with effective coping mechanisms. Besides, assigning teaching assistants or co-teachers shall alleviate the challenges of emotionally demanding classrooms, whereas workload redistribution and stress management strategies, such as adaptive scheduling to ease workloads for teachers showing early signs of burnout and flexible teaching methods that encourage natural emotional expression, need to be adopted to reduce burnout risks of EFL instructors in China.

*Global insights:* Schools in high-power-distance cultures (e.g., East Asia) should prioritize autonomy to counteract the negative effects of SA.

• **Encouraging DA to enhance emotional wellbeing of Chinese EFL instructors:**

*Current findings:* DA mitigates the impact of SA (β = 0.5505, *p* < 0.001) and correlates less harmfully with TB (*r* = 0.8421).

*Policy recommendations for China:* This study finds that implementing training programs on emotion modulation and deep acting should equip teachers with strategies to align their emotions authentically with their teaching roles. Like, workshops focusing on empathy training, role-playing, and emotional perspective-taking, along with simulated classroom experiences, often help educators to practice DA techniques effectively. Also, designing an emotionally intelligent curriculum that promotes engaging teaching methods—such as storytelling, humor, and active learning, can reduce reliance on scripted emotional responses. Besides, longitudinal tracking of DA efficiency through long-term studies should be useful top monitor EFL instructors' emotional wellbeing and refine DA-based interventions as needed.

*Global insights:* DA aligns with global education trends toward student-centered learning, while reducing reliance on performative emotions.

• **Enhancing EI to mitigate burnout among EFL instructors:**

*Current findings:* EI buffers the impact of SA (β = 1.0569, *p* < 0.001), yet excessive EI can backfire (β = −2.5361, *p* < 0.001).

*Policy recommendations for China:* This study promotes the importance of integrating EI training into teacher development programs to reduce TB. To achieve this, schools should incorporate EI assessments into teacher hiring and training processes to identify and address emotional competencies early. They can develop targeted professional development modules focused on enhancing EI skills, such as stress management and adaptive coping strategies, whereas they need to utilize AI-powered tools, including digital coaching platforms and real-time emotion recognition systems, to provide personalized EI training for educators. This study calls on human resource managers at academic institutions to implement peer mentorship programs by pairing experienced teachers with high EI to support newcomers in developing emotional resilience. Thus, with a structured yet adaptable approach, institutions across China can develop a more emotionally resilient teaching workforce, ultimately reducing turnover.

*Global insights:* Balance EI training with boundary-setting skills to prevent over-identification with students' emotions.

• **Addressing emotional exhaustion through institutional support:**

*Current findings:* SA drives emotional exhaustion (β = 0.7619, *p* < 0.001), thus fuelling burnout of EFL instructors.

*Policy recommendations for China:* To address TB, schools should implement comprehensive support systems, including wellbeing committees and anonymous stress reporting tools, to monitor emotional exhaustion proactively. Classroom policies must be optimized by integrating mental health breaks and reducing teacher-student ratios to alleviate strain. Additionally, developing positive work environments through enhanced staff recognition programs and paid mental health days can boost job satisfaction and resilience. These measures, which should be rooted in early intervention, workload management, and institutional appreciation, can mitigate emotional exhaustion, improve retention, and create a sustainable teaching workforce.

*Global insights:* Policies like Spain's *right to disconnect* laws can be effective to protect teachers from after-hours work stress.

• **Framing data-driven decision-making in teacher wellbeing:**

*Current findings:* SEM models confirm robust predictive power (CFI = 0.9844, GFI = 0.9811, RMSEA = 0.105).

*Policy recommendations for China:* To proactively address teacher burnout, institutions should adopt AI-driven and data-informed strategies. Like, they can deploy machine learning models to analyze emotional distress patterns, thus enabling early intervention through predictive analytics. They can institutionalize longitudinal studies via annual wellbeing surveys to assess EL trends and adapt policies using data-driven risk assessments. Moreover, this study proposes institutions to integrate AI-powered classroom tools, such as virtual teaching assistants for routine tasks and mental health chatbots for real-time emotional support, to reduce educators' workload. These recommendations combine predictive modeling, continuous monitoring, and intelligent automation to create a sustainable support ecosystem that mitigates burnout while enhancing teacher resilience and effectiveness.

*Global insights:* Combine data tools with human oversight to avoid privacy concerns (e.g., GDPR compliance in the EU).

• **Lemmas** ***L*_**1**_** **and** ***L*_2_-induced insights:** The results of moderation analyses for *L*_1_ and *L*_2_ carry important implications for diverse stakeholders in the educational ecosystem in China. Like, when instructors habitually reinterpret emotionally taxing situations, this study finds the adverse effects of SA on burnout to be attenuated. This finding supports their long-term psychological resilience. Conversely, reliance on ES intensifies the strain of SA, thus amplifying exhaustion and detachment. Thus, EFL instructors in China should be encouraged to consciously cultivate adaptive strategies that sustain their wellbeing.

For academic administrators, current results shows the importance of investing in professional development programs that train educators in constructive emotion regulation practices, particularly cognitive reappraisal. Therefore, institutional support structures, such as reflective practice workshops, peer mentoring, and stress management resources, can be useful to reduce the reliance on maladaptive suppression while reinforcing adaptive reappraisal. Moreover, this study pleads policymakers to formally recognize EL as an integral component of teaching. Embedding teacher wellbeing into national quality standards and accreditation frameworks shall ensure that ER support becomes a systemic rather than individual responsibility.

#### 6.2.1 Summary

To build an effective support system for EFL instructors, this study pleads stakeholders to pursue multi-level interventions that extend across policy, technological, and institutional domains, while also embedding resilience-building measures into teacher training and professional development programs. Like, at the policy level, governments can introduce regulatory safeguards, such as limits on teaching hours and earmarked investments in teacher mental health programs. The technological innovations, including wearable stress monitors and AI-enabled assessment platforms, need to be integrated into training curricula to help future instructors to monitor and reflect their wellbeing in real time. At the institutional level, schools can cultivate psychologically safe environments, where EFL instructors openly address emotional challenges and engage in collaborative peer-support practices.

Crucially, this study determines that the management of emotional demands is non-uniform in nature and varies with instructors' ER strategies. Thus, any professional development programs should skilfully frame adaptive strategies, like CR, while equipping teachers to avoid reliance on maladaptive approaches, such as ES. Especially, embedding structured ER training modules into teacher preparation and in-service development programs enable EFL instructors to better navigate affective demands throughout their careers. By aligning policy safeguards, innovative monitoring technologies, and institutional cultural change with long-term capacity-building in teacher education, stakeholders can reduce burnout while cultivating a sustainable and future-ready educational ecosystem in China and beyond.

### 6.3 Generalizability of insights

The EL is a fundamental occupational demand. Thus, aforementioned insights can hold broad relevance for teaching professionals worldwide. Their generalizability beyond Chinese EFL instructors is supported by universal psychological mechanisms, like ER, authenticity in emotional displays, and individual EI, which operate consistently across diverse cultures and institutions. Evidences from real-world cases also attest those as follows:

#### 6.3.1 Corroboration in real-world

A number of real-world cases provide corroborating evidence for current insights. First, the protective role of ER in reducing TB resonates with the wellbeing programs for teachers across cultures. For example, the United Kingdom piloted nationwide initiatives promoting mindfulness-based stress reduction in schools, which showed measurable reductions in teacher stress and improvements in job satisfaction. These outcomes parallel current findings that ER mitigates TB, thereby implying that interventions, such as mindfulness and acceptance-based coping, have universal value when adapted to cultural context. In the United States, school districts adopting the social and emotional learning frameworks for educators reported an enhanced teacher resilience, which also aligned with current result that the ER training would buffer against emotional exhaustion.

Second, the detrimental effects of SA are hardly unique to China but observed globally. For example, research on teachers in Finland indicated that pressure to maintain a consistently cheerful demeanor in the classroom contributed to emotional exhaustion and attrition, even within a supportive welfare-state context. In Japan, where strict norms of emotional display were ingrained in the teaching profession, a reliance on SA could be linked to the karoshi, the work-related exhaustion and burnout. This supported that inauthentic emotional displays would intensify stress across high-demand educational systems. These cases reinforce the generalizability of current insights that the SA positively correlates with TB.

Third, this study finds that DA serves as a healthier alternative aligns with practices in student-centered systems worldwide. In Scandinavian countries, where teachers are usually encouraged to align their emotions authentically with pedagogical practices through approaches, such as the fika, structured relational breaks, the DA strategies contributed to improved teacher-student relationships and lower burnout. Likewise, in Canadian schools, professional development workshops emphasizing empathy, perspective-taking, and storytelling as instructional strategies could considerably help their teachers to internalize authentic emotional engagement. This scenario echoed that DA reduces reliance on surface-level displays of emotions.

Fourth, the role of EI as a moderator finds cross-cultural support. Like, in South Korea, teacher-training programs increasingly incorporated EI assessments to ensure that new hires would possess sufficient capacity for stress regulation in demanding classroom environments. Comparable initiatives in Australia employ the coaching platforms to strengthen EI skills among early-career teachers, thus resulting in reduced attrition rates. These global practices reflect a current insight that EI buffers the negative effects of SA, even while excessive emotional over-identification being counter-productive.

Moreover, the need for institutional-level support to counter emotional exhaustion has been recognized internationally. Spain's right to disconnect-legislation, which limited after-hours professional obligations, could provide a structural buffer against TB. In France, policies mandating reduced class sizes in early grades served as systemic interventions to manage workload and emotional strain. These cases illustrate that, as in China, institutional support mechanisms are crucial to complement individual-level strategies, such as ER, DA, and EI training. Therefore, the proposed mechanisms resonate with international evidence on teacher wellbeing. What differs is the ways in which interventions must be tailored to diverse cultures and institutions.

#### 6.3.2 Cultural specificity

The proposed mechanisms, namely the protective role of ER, the detrimental effects of SA, the relative benefits of DA, and the buffering function of EI, are theoretically robust and supported by broader EL literature. However, their expression is likely to vary across cultural and institutional contexts. For example, in high power-distance, exam-oriented systems like China, the reliance on SA can be heightened by hierarchical expectations and rigid norms, whereas in low power-distance contexts, such as Northern Europe, greater teacher autonomy can reduce the need for inauthentic displays. Likewise, collectivist cultures tend to emphasize institution-driven wellbeing initiatives, while individualist cultures often privilege self-directed coping strategies.

These distinctions show that although the relationships tested in this study are generalizable in principle, the intensity and manifestation of their effects are contingent on cultural norms of emotional expression, institutional autonomy afforded to teachers, and the broader educational environment. Thus, in future, this study plans to extend the present model by testing this with cross-cultural samples of teachers, both within and beyond EFL contexts, to further validate its applicability and refine context-specific interventions.

## 7 Conclusions

The present study examined the complex issue of academic burnout among EFL instructors by examining how EL strategies, specifically SA and DA, would interact with emotional competencies, such as ER and EI, to either exacerbate or mitigate their burnout. To address why some instructors experienced burnout while others often thrived in demanding environments, this study investigated the impact of different EL strategies on teachers' emotional wellbeing. In particular, this study compared the effects of DA and SA by assessing whether DA could serve as a more effective strategy for reducing burnout than SA. Focused on the high-stress educational context of China, this examined the protective function of emotional competencies in safeguarding instructors from the deleterious effects of significant and persistent emotional demands. Specifically, this explored the mechanisms through which emotional competencies could influence the relationship between EL and TB, while focusing on the mediating role of ER and the moderating role of EI in the relationship between SA and TB. Subsequently, this conducted key statistical analyses using research data of 448 EFL instructors in China with a questionnaire recovery rate of 93.08%, and thus tested the validity of five proposed hypotheses. Current results determined that the ER could significantly reduce the TB of EFL instructors in China (β = 0.1447, *p* < 0.001), whereas SA strongly contributed to TB (β = 1.5617, *p* < 0.001). The DA had a weaker impact on burnout compared to SA (β = 0.6298), which implied that DA mitigated teachers' emotional dissonance. The mediation analysis found that the SA negatively influenced ER, which would increase burnout (indirect effect = 0.4965, 95% CI [0.4190, 0.5805]). The EI moderated the impact of SA on TB (β = 4.0092, *p* < 0.001).

A key contribution of this study was to position the curvilinear moderating role of EI in the relationship between SA and TB of EFL instructors. This study promoted the role of DA over SA, while advocating for peer support systems and regular assessments of psychological wellbeing to prevent burnout. To mitigate TB among Chinese EFL instructors, educational institutions should integrate ER and EI training to help instructors managing emotional demands effectively. This study pleaded administrators to encourage DA for reducing emotional dissonance and lower burnout, whereas schools can establish peer support networks and counseling services to enhance resilience. Also, periodic psychological assessments should be conducted to detect early burnout signs for timely intervention. Thus, to prevent excessive emotional labor and ensure a balanced teaching environment, workload policies must be revised. Besides, institutional policies can prioritize mental wellbeing of instructors by developing a supportive work culture, which is fundamental to enhance the job satisfaction and promote sustainable teaching practices.

### 7.1 Limitations and future research scopes

The present study has several limitations that must be acknowledged, along with corresponding avenues for future research. Like, the EI buffered the negative impact of SA, even though its curvilinear effect (β = −2.5361, *p* < 0.001) indicated that excessive EI paradoxically undermined the wellbeing of Chinese EFL instructors. This complex relationship warrants further investigation, particularly with regard to potential thresholds of EI and the risks associated with emotional over-engagement. Future researchers can also employ experimental designs to determine the optimal levels and training intensities of EI that develop a sustained resilience among EFL instructors in China and beyond. Second, the current insights emphasized the individual-level factors, such as ER and EI, without accounting for institutional-level drivers, like class size, leadership style, or organizational climate. Thus, in future, one should employ multi-level models to investigate how school- and system-level policies moderate the effects of SA and DA on burnout dynamics.

Third, the demographic imbalances within current dataset need to be recognized as potential influences on the observed patterns of ER, EL, and EI. Thus, those provided a crucial context for interpreting the proposed insights with appropriate caution. Fourth, the cross-sectional design limited the ability of this study to draw causal inferences. To address this, future researchers can adopt the longitudinal approaches to capture temporal dynamics in ER, EL strategies, and EI over time. This approach enables examination of whether teachers, who initially rely on SA, gradually shift toward DA, and whether ER strengthens or diminishes with accumulated professional experience. Besides, longitudinal data can yield some critical periods during which targeted interventions are most impactful, thereby deriving valuable guidance in developing sustained support systems for teachers.

Fifth, one needs to explore the role of contextual moderators shaping the SA–TB association and the role of ER. Factors, such as autonomy, workload intensity, and psychological safety, can indicate how SA translates into burnout. Also, leadership support and collegial climate often strengthen the adaptive ER and regulate any maladaptive strategies. Thus, integrating organizational resources with individual ER in future research shall provide a more systemic understanding of burnout trajectories among instructors. Sixth, the cultural specificity of this study needs to be considered elaborately in future. Since current research data were drawn exclusively from Chinese EFL instructors, the insights of this study fail to reflect the contextual features of China's high power-distance environment and norms of emotional restraint, which often intensify the reliance on SA. Therefore, to strengthen external validity in future studies, one should incorporate cross-cultural comparisons, such as between Scandinavian and Asian contexts, and employ qualitative methods to capture the cultural contexts in EL.

Moreover, current reliance on self-reported data introduces risks of bias, including CMB and social desirability effects. Thus, future researchers need to adopt mixed-method approaches that integrate self-reports with objective indicators, such as wearable stress sensors, physiological markers, or administrative data on absenteeism and attrition. They can incorporate multi-source or multi-method designs, such as combining survey data with classroom observations or performance records, to triangulate the findings and further reduce CMB-related concerns. Besides, emerging interventions, including AI-driven emotional support systems and predictive analytics for teacher wellbeing, should be empirically tested across diverse educational systems to evaluate the corresponding culture-specific and culture-independent effectiveness.

## Data Availability

The raw data supporting the conclusions of this article will be made available by the authors, without undue reservation.
